# MHC class II antigen presentation pathway in murine tumours: tumour evasion from immunosurveillance?

**DOI:** 10.1054/bjoc.2000.1415

**Published:** 2000-11

**Authors:** W Walter, K Lingnau, E Schmitt, M Loos, M J Maeurer

**Affiliations:** Department of Medical MicrobiologyInstitute for Immunology, Johannes Gutenberg University, Mainz, D-55101, Germany

**Keywords:** MHC, gene regulation, transcription factors, antigen processing

## Abstract

Qualitative differences in the MHC class II antigen processing and presentation pathway may be instrumental in shaping the CD4+ T cell response directed against tumour cells. Efficient loading of many MHC class II alleles with peptides requires the assistance of H2-M, a heterodimeric MHC class II-like molecule. In contrast to the *HLA-DM* region in humans, the β-chain locus is duplicated in mouse, with the *H2-Mb1* (*Mb1*β-chain distal to *H2-Mb2 (Mb2)* and the *H2-Ma* (*Ma*) α-chain gene). Here, we show that murine *MHC* class II and *H2-M* genes are coordinately regulated in murine tumour cell lines by T helper cell 1 (IFN-γ) and T helper cell 2 (IL-4 or IL-10) cytokines in the presence of the MHC class II-specific transactivator CIITA as determined by mRNA expression and Western blot analysis. Furthermore, Mαβ1 and Mαβ2 heterodimers are differentially expressed in murine tumour cell lines of different histology. Both H2-M isoforms promote equally processing and presentation of native protein antigens to H2-A^d^- and H2-E^d^-restricted CD4+ T cells. Murine tumour cell lines could be divided into three groups: constitutive MHC class II and CIITA expression; inducible MHC class II and CIITA expression upon IFN-γ-treatment; and lack of constitutive and IFN-γ-inducible MHC class II and CIITA expression. These differences may impact on CD4+ T cell recognition of cancer cells in murine tumour models. © 2000 Cancer Research Campaign


*British Journal of Cancer* (2000) **83**(9), 1192–1201
© 2000 Cancer Research Campaign
doi: 10.1054/ bjoc.2000.1415, available online at http://www.idealibrary.com on IDEAL®

### MHC class II antigen presentation pathway in murine tumours: tumour evasion from immunosurveillance?

**W Walter[1], K Lingnau[2], E Schmitt[2], M Loos[1] and MJ Maeurer[1]**

[1]Department of Medical Microbiology, [2]Institute for Immunology, Johannes Gutenberg University, D-55101 Mainz, Germany

**Summary** Qualitative differences in the MHC class II antigen processing and presentation pathway may be instrumental in shaping the CD4+ T cell response directed against tumour cells. Efficient loading of many MHC class II alleles with peptides requires the assistance of H2-M, a heterodimeric MHC class II-like molecule. In contrast to the *HLA-DM* region in humans, the β-chain locus is duplicated in mouse, with the *H2-Mb1* (*Mb1*β-chain distal to *H2-Mb2 (Mb2)* and the *H2-Ma* (*Ma*) α-chain gene). Here, we show that murine *MHC* class II and *H2-M* genes are coordinately regulated in murine tumour cell lines by T helper cell 1 (IFN-γ) and T helper cell 2 (IL-4 or IL-10) cytokines in the presence of the MHC class II-specific transactivator CIITA as determined by mRNA expression and Western blot analysis. Furthermore, Mαβ1 and Mαβ2 heterodimers are differentially expressed in murine tumour cell lines of different histology. Both H2-M isoforms promote equally processing and presentation of native protein antigens to H2-A$^d$- and H2-E$^d$-restricted CD4+ T cells. Murine tumour cell lines could be divided into three groups: constitutive MHC class II and CIITA expression; inducible MHC class II and CIITA expression upon IFN-γ-treatment; and lack of constitutive and IFN-γ-inducible MHC class II and CIITA expression. These differences may impact on CD4+ T cell recognition of cancer cells in murine tumour models. © 2000 Cancer Research Campaign

**Keywords**: MHC; gene regulation; transcription factors; antigen processing

Most of the tumour antigens defined by murine or human T cells represent targets for major histocompatibility complex (MHC) class I restricted CD8+ T cells. Nevertheless, recent data (reviewed in Pardoll and Topalian, 1998) rekindled interest in CD4+ T cells mediating anti-tumour-directed immune responses. Both, CD4 and CD8 T cell epitopes may have to be incorporated in a tumour vaccine in order to induce a strong and effective cellular immune response. Tumour-specific T cells may directly recognize MHC class II-positive tumour cells (Mongini et al, 1996; Armstrong et al, 1997), or they may confer protective immunity on cancer cells in the absence of MHC class II molecules (Ossendorp et al, 1998). Thus, efficient MHC class II restricted presentation of tumour-associated antigens, either by tumour cells themselves, or alternatively by professional antigen-presenting cells, appears instrumental in initiating CD4+ T cell responses.

Major histocompatibility complex (MHC) class II molecules are heterodimeric (αβ) cell surface glycoproteins which present peptides derived from self or foreign antigens to CD4+ T cells. Newly synthesized MHC class II α and β chains assemble in the endoplasmic reticulum (ER) with a third glycoprotein, the invariant chain (Ii), to nonameric $(\alpha\beta\text{-Ii})_3$ complexes (Cresswell, 1996). These nonamers are targeted by signals within the cytoplasmic domain of the Ii (Bakke and Dobberstein, 1990) to specialized compartments of the endocytic pathway termed MIICs (MHC class II compartments), where peptide loading occurs (Kleijmeer et al, 1997). During transport, Ii is stepwise proteolytically cleaved (Cresswell, 1998) yielding a nested set of Ii derived peptides, termed CLIP, for MHC class II-associated invariant peptides, which occupy the peptide-binding groove of class II αβ dimers (Riberdy et al, 1992). CLIP is subsequently exchanged for tightly bound antigenic peptides derived from internalized antigens or endogenous proteins (Wolf and Ploegh, 1995).

Although exchange of CLIP for antigenic peptides might occur spontaneously in MHCIIs at lysosomal-like pH (Urban et al, 1994), H2-M/DM is required for that final step of peptide loading by many MHC class II alleles (Denzin et al, 1994; Miyazaki et al, 1996; Wolf et al, 1998). H2-M/DM has been shown to interact with MHC class II in MIICs (Sanderson et al, 1996) and to catalyze the exchange of CLIP for cognate peptides by facilitating the removal of CLIP and stabilizing the transiently peptide-free state of MHC class II molecules following CLIP release (Denzin et al, 1996). Simultaneously, H2M/DM functions as a peptide editor, which serves to positively select peptides that can stably bind to MHC class II molecules (Van Ham et al, 1996). Thus, expression of H2-M/DM molecules may critically affect the peptide repertoire displayed to the T cell compartment. In contrast to the *HLA-DM* loci in humans, the murine *H2-M* region encoded within the MHC contains one *Ma* gene, but two *Mb* genes, termed *Mb1* and *Mb2* (Cho et al, 1991; Kelly et al, 1991). Regulation of MHC class II genes occurs primarily at the transcriptional level (Mach et al, 1996) and is controlled by a non-DNA-binding cofactor, the class II *trans*-activator CIITA (Mach et al, 1996). Until this end, the potential role of a differential *Mb1* or *Mb2* expression in professional or non-professional APCs (i.e. tumour cells) has not been addressed.

Here, we investigated the regulation of *H2-M*, *MHC* class II and *CIITA* gene expression in murine tumour cells lines by Th1 (IFN-γ)- and Th2 (IL-4 or IL-10) cytokines. In addition, we examined whether *Mb1* and *Mb2*, and by consequence Mαβ1 and Mαβ2 heterodimers, are differentially expressed in different tumour

*Received 25 February 2000*
*Accepted 21 June 2000*

*Correspondence to:* MJ Maeurer types and if they are able to effectively present surrogate target antigens to MHC class II-restricted T cell lines.

### MATERIALS AND METHODS

### Animals

C57BL/6 mice ($H2^b$) and New Zealand White rabbits were purchased from Charles River Laboratories (Sulzfeld, Germany). TCR DO11.10 transgenic mice expressing a TCR-α/β specific for peptides 323–339 from ovalbumin presented by H2-$A^d$ (Murphy et al, 1990) and mice expressing a TCR-α/β specific for peptides 111–119 from influenza haemagglutinin (HA) presented by H2-$E^d$ (Kirberg et al, 1994) were generous gifts from Dr D Loh and Dr J Kirberg at the Basel Institute for Immunology (Basel, Switzerland).

### Cell lines and culture conditions

Murine cell lines included: Renca ($H2^d$) renal adenocarcinoma kindly provided by Dr B Seliger (University of Mainz, Germany), P815 ($H2^d$) mastocytoma, TS/A ($H2^d$) mammary adenocarcinoma, MC-38 ($H2^b$) colon adenocarcinoma, MCA-102 ($H2^b$) fibrosarcoma, L929 ($H2^b$) fibroblasts, B16 ($H2^b$) melanoma, RMA ($H2^b$) T cell lymphoma, RMAs ($H2^b$) T cell lymphoma, EL-4 ($H2^b$) thymoma, YAC-1 ($H2^a$) T cell leukaemia, P388D1 ($H2^d$) macrophages, IC-21 ($H2^b$) macrophages, A20 ($H2^d$) B cell lymphoma (kindly provided by Dr G Hämmerling, DKFZ, Heidelberg, Germany) and LB27.4 ($H2^d$) B cell lymphoma. Cell lines were maintained in RPMI-1640 (Gibco, Eggenstein, Germany) supplemented with 10% heat-inactivated fetal bovine serum, 2 mM L-glutamate, 100 IU $ml^{-1}$ penicillin, 100 μg $ml^{-1}$ streptomycin (all from Gibco) and 50 μM 2-mercaptoethanol (Sigma, Deisenhofen, Germany), termed complete medium (CM). In experiments using rIL-4 (a generous gift from Shering Ploegh, Kenilworth NJ, USA) rIL-10 or rIFN-γ (both from PharMingen, Hamburg, Germany), culture medium was changed before experiment and IL-4 (250 U $ml^{-1}$), IL-10 (100 ng $ml^{-1}$) or IFN-γ (150 U $ml^{-1}$) were added for 72 h.

### Preparation of CD4+ T cells

Isolation of CD4+ Mel-$14^{high}$ naive T cells from spleens of TCR transgenic mice and generation of CD4+ Th cells that express a Th1 cytokine pattern after specific activation with antigen, were performed as recently described in detail elsewhere (Lingnau et al, 1998).

### Antibodies

The anti-class II mAbs FITC-conjugated 39–10–8 (anti-H2-$A^d$) and PE-conjugated 14–4–4S (anti-H2-$E^{k,d}$) were obtained from PharMingen; FITC-conjugated secondary staining reagents: goat anti-hamster IgG, rabbit anti-rat IgG, goat antimouse IgG and goat anti-rabbit IgG were purchased from Dianova (Hamburg, Germany) and unlabelled, FITC- or PE-conjugated isotype-matched control antibodies were from Coulter-Immunotech (Hamburg, Germany). The rabbit antisera R.Mα-C.69.3, R.M-β1/2–C.71.3 and R.hCLIP73.11 were prepared by immunizing rabbits with carboxy-terminal peptides from H2-Mα (aa 238–248) or H2-Mβ1/2 (aa 228–243) coupled by an added amino terminal cysteine to diphtheria toxoid-MCS (Chiron Mimotopes, Victoria, Australia). Antisera were affinity purified using columns of NHS-activated Fast Flow Sepharose 4 (Pharmacia, Freiburg, Germany) cross-linked to the respective Mα, Mβ or CLIP peptide according to the manufacturer's instructions. Individual fractions of affinity purified antisera were screened for specific antibody titres by ELISA and Western blot analysis using T2 cell lines transfected either with *Ma*, *Mb1* or *Mb2* genes (Walter et al, in press).

### Template cDNA preparation and competitive reverse transcription PCR

Total RNA isolation and cDNA synthesis have been previously described (Walter et al, 1996). Briefly, PCR amplification was performed in an amplification mix adjusted to 50 μl containing 50–100 ng cDNA, 10 mM Tris-HCl (pH 8.3), 50 mM KCl, 1.5 mM $MgCl_2$, 0.01 (w/v) gelatin, 1 mM each dNTP, 25 pmol each primer and 2.5 U of Ampli Taq Gold Polymerase (Perkin-Elmer, Weiterstadt, Germany). The RT-PCR amplification profile involved an initial denaturation step at 94°C for 10 min followed by 35 cycles of 94°C for 1 min, 62°C for 1 min and 72°C for 1 min; the last extension was for 10 min at 72°C. The following primers were used for PCR: β-actin sense 5′-TGGAATCCT-GTGGCATCCATGAAAC-3′; β-actin antisense 5′-TAAAAC GCAGCTCAGTAACAGTCCG-3′; Ma sense 5′-AAGGTATG-GAGCATGAGCAGAAGT-3′; Ma antisense 5′-GATCAGTCAC-CTGAGCACGGT-3′; two sets of primers were used to amplify CIITA: sense 5′-CCATCTTGCGCCCGCAGGCT-3′; antisense 5′-GTGCCAGGCTCTTGGCTCC-3′; and a set previously identified to amplify both human and mouse CIITA (Chang et al, 1996). Competitive RT-PCR analysis has been performed as previously described (Bouaboula et al, 1992). After reverse transcription, cDNA concentrations were normalized by co-amplifying constant amounts of cDNA (50 ng) and known concentrations of the serially diluted β actin competitor plasmid pMCQ (kindly provided by Dr Blankenstein, University of Berlin, Germany). PCR products generated by the competitor plasmid pMCQ (248 bp) and the endogenous *β-actin* mRNA (348 bp) were resolved on ethidium-bromide-stained agarose gels and band intensities were quantified as $OD_{302}$ units by means of the Gelprint 2000i densitometer (MWG Biotech, Ebersberg, Germany). The pMCQ concentration which was required to achieve equal band intensities for both fragments was determined by linear regression analysis and the number of copies of *β-actin* mRNA molecules per μg of total RNA for each cDNA sample calculated (Bouaboula et al, 1992). For semiquantitative analysis of *Ma* mRNA expression, a heterologous Ma competitor fragment (termed Ma cf) was generated as described according to MIMIC PCR technology (Haberhausen et al, 1998). Following co-amplification, Ma cf yields a 742 bp fragment as compared to the 794 bp PCR product derived from endogenous *Ma* mRNA. Equivalent to *β-actin* mRNA analysis, the number of copies of *Ma* mRNA molecules per μg of total RNA were determined for each sample by titration of known amounts of the serially diluted Ma cf against constant amounts of cDNA (100 ng). In order to compare differences in *Ma* mRNA expression levels, samples of each cell line were normalized for cDNA contents based on the number of *β-actin* mRNA molecules. Expression of *Ma* transcripts in cytokine-treated samples of each

© 2000 Cancer Research Campaign individual cell line was calculated as *Ma* mRNA in cytokine-treated cells/*Ma* mRNA expression (= 1) in non-treated cells.

### Ratio reverse transcriptase PCR analysis

The ratio reverse transcriptase PCR (ratio-RT-PCR) assay performed in this study was based on the simultaneous amplification of *Mb1* and *Mb2* transcripts using primers (Mb1/2 sense 5′-GGACCATGGCTGCACTCTGGC-3′, Mb1/2 antisense 5′-GCATCACGGGCTCCCTTGTGT-3′) annealing within conserved regions (exon 1 and 3) of both *Mb* messages (Walter et al, 1996). Equal amplification efficiency of both *Mb* transcripts was assured by comparative cycle kinetic and linear regression analyses (Bouaboula et al, 1992) using cloned *Mb1* and *Mb2* full-length cDNA (Walter et al, 1996). Discrimination between co-amplified *Mb* transcripts was performed utilizing *Mb1*- and *Mb2*-specific restriction sites within exon 2. Based on the polymorphism of *Mb1* and *Mb2* genes (Walter et al, 1996), we took advantage of the restriction enzymes *Eae*I and *Xba*I for discrimination of $Mb1^d$ and $Mb2^d$ transcripts, respectively. *Bam*HI, which cleaves at different sites within both $Mb^{a,b}$ targets, was implemented to discriminate between $Mb1^{a,b}$ and $Mb2^{a,b}$ messages. PCR was performed using Ampli Taq Gold Polymerase and standard PCR conditions. After 25 cycles, the amplified mixture was diluted 25-fold in fresh PCR amplification mixture containing 25 nCi $\mu l^{-1}$ [$\alpha$-$^{32}$P]dCTP (ICN, Eschwege, Germany) and then two additional cycles were performed. The labelled *Mb* PCR products, or their respective restriction fragments, were separated on 6% polyacrylamide gels. For quantification of individual *Mb* fragments, gels were subjected to autoradiography. Corresponding bands were excised from the gel and radioactivity was measured by means of a β-counter (LS6000TA, Beckman, München, Germany) using a Cerenkov program. In order to calculate the ratio of *Mb1* and *Mb2* expression levels, their respective restriction fragments were corrected for length and cytosine and guanine (GC) content, since exclusively dCTP was radioactively labelled in the assay.

### Western blot analysis

Cells were lysed at $1 \times 10^7$ cells $ml^{-1}$ in 20 mM Tris-HCl (pH 7.4) buffer containing 1% NP40 (Sigma), 5 mM $MgCl_2$, 5 μg $ml^{-1}$ chymostatin, 2.5 μg $ml^{-1}$ leupeptin, 5 μg $ml^{-1}$ pepstatin A, 200 μM PMSF (all protease inhibitors were from Boehringer) for 30 min at 4°C. Nuclei and insoluble debris were removed by centrifugation at 14 000 rpm for 30 min. Aliquots of $1–5 \times 10^6$ cell equivalents of a cleared lysate were mixed with Laemmli buffer, boiled for 5 min, separated on 10% polyacrylamide SDS-gels, and then transferred onto Immobilon PVDF membranes (Millipore, Eschborn, Germany). Membranes were blocked overnight in blocking reagent (Boehringer). Antibody binding was detected by incubation with horseradish peroxidase-conjugated goat anti-rabbit immunoglobulin (Dianova) followed by enhanced chemiluminescence using Super-Signal-Ultra (Pierce, Rockford IL, USA).

### Flow cytometry

Cells ($5 \times 10^5$ per sample) were washed in PBS supplemented with 1% BSA (PBS/BSA) and incubated on ice with FITC- or PE-conjugated or unlabelled primary mAb for 30 min. After washing in PBS/BSA, cells were either fixed in 1% formaldehyde for flow cytometry, or, for unlabelled primary mAb, a secondary staining reagent, FITC-goat anti-hamster IgG (FITC-GAH) or FITC-goat anti-mouse IgG (FITC-GAM) or FITC-rabbit anti-rat IgG (FITC-RAR) or FITC-goat anti-rabbit IgG (FITC-GAR) was applied for 30 min at 4°C. Background fluorescence was evaluated using either FITC-or PE-coupled irrelevant matched isotypes, or unlabelled matched isotypes and FITC-GAH, FITC-GAM, FITC-RAR or FITC-GAR. Cell surface fluorescent labelling was visualized on an EPICS®-PROFILE II flow cytometer (Coulter Immunotec Co, Hamburg, Germany) and data analysis was performed using EPICS®ELITE 3.0 software.

### Antigen presentation assay

Presentation assays were performed by incubating A20 B cells or IFN-γ-stimulated (150 U $ml^{-1}$ for 48 h) P388D1 macrophages as APCs ($5 \times 10^4$ cells per well), T cells specific for $OVA_{323–339}$ presented by H2-$A^d$ or T cells specific for $HA_{111–119}$ presented by H2-$E^d$ (both at $2 \times 10^5$ cells per well), and varying concentrations of antigen in flat-bottomed 96-well plates in a final volume of 200 μl per well. Supernatants were collected after 24 h and assayed for IFN-γ by ELISA as recently described (Lingnau et al, 1998). Alternatively, A20 and P388D1 cells were fixed in 1% paraformaldehyde at room temperature for 20 min and washed extensively with CM prior to assay.

### RESULTS

### Differential expression of *MHC* class II and *CIITA* in IL-4, IL-10 or IFN-γ-stimulated tumour cell lines

In order to examine the regulation of genes involved in the MHC class II antigen processing and presentation pathway, we defined the status of MHC class II cell surface (H2-A and H2-E) and *CIITA* mRNA expression in a panel of murine tumour cell lines prior to and after stimulation with IL-4, IL-10 or IFN-γ. Table 1 summarizes the mean values of fluorescence obtained for H2-A and H2-E expression correlated with specific *CIITA* transcripts in IL-4, IL-10 or IFN-γ-treated cells. Tumour cell lines could be divided into three groups based on MHC class II cell surface expression. The first group is defined by constitutive MHC class II and CIITA expression and includes the B cell lymphoma cell lines A20 and LB27.4, as well as the macrophage cell line P388D1. Different cytokines were found to enhance H2-A and H2-E cell-surface expression dependent on the cell type: IL-4 represents the most potent stimulus for the B-cell lymphoma cell lines and IFN-γ for P388D1 macrophages. Remarkably, stimulation of P388D1 cells with IL-10 did not affect steady state H2-A surface expression level, but resulted in a reduced number of detectable H2-E surface molecules as compared to P388D1 cells cultured in medium without cytokine addition. The second group exhibits no constitutive, but inducible expression of *MHC* class II and *CIITA* genes upon IFN-γ-treatment, represented by the macrophage-derived cell line APC IC-21, the melanoma cell line B16, L929 fibroblasts and the renal adenocarcinoma cell line Renca. Of note, although IFN-γ-treatment of Renca cells did not induce appreciable H2-A or H2-E surface expression, RT-PCR analysis of *H2-Aa*, *-Ab*, *-Ea* and *-Eb* mRNA expression demonstrated the presence of specific mRNA for all components of the MHC class II antigen presentation pathway in concert with *CIITA* mRNA after

© 2000 Cancer Research Campaign

**Table 1** Expression of cell surface MHC class II Ags and CIITA mRNA in cytokine-treated professional and nonprofessional APCs

| | | H2-A surface expression | | | | H2-E surface expression | | | | *CIITA* mRNA expression | | | |
|---|---|---|---|---|---|---|---|---|---|---|---|---|---|
| **Cell-line** | **Histology** | **Nil** | **IL-4** | **IL-10** | **IFN-γ** | **Nil** | **IL-4** | **IL-10** | **IFN-γ** | **Nil** | **IL-4** | **IL-10** | **IFN-γ** |
| A20 | B cell lymphoma | 25.54 | 57.61 | 41.28 | 31.73 | 99.75 | 165.41 | 161.71 | 128.68 | + | + | + | + |
| LB27.4 | B cell lymphoma | 16.31 | 24.18 | 19.1 | 21.45 | 26.77 | 45.32 | 39.58 | 42.89 | + | + | + | + |
| P388D1 | macrophage | 1.34 | 3.78 | 1.36 | 14.42 | 3.76 | 11.67 | 2.72 | 51.93 | + | + | + | + |
| IC-21 | macrophage | 0.59 | 0.74 | 0.56 | 2.62 | n.d. | n.d. | n.d. | n.d. | – | – | – | + |
| B16 | melanoma | 0.23 | 0.51 | 0.97 | 8.57 | n.d. | n.d. | n.d. | n.d. | – | – | – | + |
| L929 | fibroblast | 0.61 | 0.65 | 0.58 | 2.89 | n.d. | n.d. | n.d. | n.d. | – | – | – | + |
| Renca | renal cell adenocarcinoma | 0.60 | 0.73 | 0.89 | 0.75 | 0.82 | 0.91 | 0.78 | 0.87 | – | – | – | + |
| P815 | mastocytoma | 0.88 | 0.96 | 0.77 | 0.72 | 0.87 | 0.92 | 0.83 | 0.74 | – | – | – | – |
| TS/A | mamma adenocarcinoma | 0.22 | 0.29 | 0.23 | 0.26 | 0.47 | 0.51 | 0.55 | 0.49 | – | – | – | – |
| MC-38 | colon adenocarcinoma | 0.45 | 0.42 | 0.57 | 0.59 | n.d. | n.d. | n.d. | n.d. | – | – | – | – |
| MCA-102 | fibroblast | 0.34 | 0.29 | 0.39 | 0.37 | n.d. | n.d. | n.d. | n.d. | – | – | – | – |
| EL-4 | thymoma | 0.12 | 0.07 | 0.08 | 0.10 | n.d. | n.d. | n.d. | n.d. | – | – | – | – |
| RMA | T cell lymphoma | 0.32 | 0.39 | 0.47 | 0.44 | n.d. | n.d. | n.d. | n.d. | – | – | – | – |
| RMAs | T cell lymphoma | 0.46 | 0.40 | 0.53 | 0.41 | n.d. | n.d. | n.d. | n.d. | – | – | – | – |
| YAC-1 | T cell lymphoma | 0.17 | 0.19 | 0.19 | 0.20 | n.d. | n.d. | n.d. | n.d. | – | – | – | – |

Cells were stained with FITC-or PE-conjugated isotype-matched irrelevant antibody (negative control), FITC-labelled mAb 39-10-8 (anti-H2-$A^d$), or PE-conjugated mAb 14-4-4S (anti-H2-$E^{k,d}$) and analysed by flow cytometry. Results are expressed as mean channel fluorescence. Expression of *CIITA* mRNA was determined by RT-PCR as described in Material and Methods. Cells were cultured in medium (Nil) or in medium supplemented with 250 U $ml^{-1}$ IL-4, 100 ng $ml^{-1}$ IL-10 or 150 U $ml^{-1}$ IFN-γ for 48 h.

treatment with IFN-γ (data not shown). The third group encompasses cell lines that lack both constitutive and IFN-γ-inducible MHC class II or CIITA expression including the mammary adenocarcinoma TS/A, the colon adenocarcinoma MC-38, the hepatic fibrosarcoma MCA-102, the mastocytoma P815 and the T cell-derived cell lines RMA, RMAs, EL-4 and YAC-1.

### Discoordinate vs coordinate expression of *H2-M* and *MHC* class II genes in tumour cells

To address the question whether *H2-M* and *MHC* class II genes are coordinately regulated in IL-4, IL-10 or IFN-γ-stimulated tumour cells, we analysed *Ma* and *Mb* mRNA expression by RT-PCR. Low levels of *Ma* and *Mb* mRNA could be detected in each cell line independent of MHC class II and CIITA expression (data not shown). In view of this and based on the observation that the human *DMA* and *DMB* genes appear to be co-regulated (Westerheide et al, 1997), we examined the regulation of *H2-M* genes by analysing *Ma* mRNA expression in untreated, IL-4-, IL-10- or IFN-γ-stimulated tumour cells utilizing a semiquantitative RT-PCR approach. In a first step, the amount of cDNA of each individual sample was evaluated using *β-actin* as a standard (Figure 1A–C), followed by determination of the *Ma* mRNA expression level (Figure 1D–F). For both steps, constant amounts of cDNA and serially diluted competitor fragments were co-amplified (Figure 1A and 1D). The concentration of the competitor required to achieve equal band intensities for both fragments was determined by linear regression analysis (Figure 1B and 1E) and the relative mRNA levels (molecules $\mu g^{-1}$ of total RNA) were calculated (Figure 1C and 1F, white bars). In order to determine differences in *Ma* mRNA expression levels, the relative amount of *β-actin* mRNA molecules was used to standardize the cDNA contents of each sample (Figure 1F, black bars). The results of the competitive RT-PCR analysis for each cell line are summarized in Table 2. Coordinate regulation of *MHC* class II and *H2-M* genes could be detected in the CIITA-positive B cell lines A20 and LB27.4. Like MHC class II, IL-4 followed by IFN-γ and IL-10 represented the most potent upregulator of constitutive *Ma* mRNA expression (Figure 1F and Table 2). Similar to its effect on MHC class II, IFN-γ induced a substantial increase of basal *Ma* mRNA expression in CIITA-positive tumour cells, including P388D1 and IC-21 macrophages, L929 fibroblasts, B16 (melanoma) and Renca (renal adenocarcinoma) cells. Enhanced *Ma* mRNA expression could also be detected in IL-4-treated P388D1 and IC21 macrophages, while IL-4 did not impact on *Ma* mRNA levels in IL-4-stimulated L929, B16 and Renca tumour cells, which lack CIITA and MHC class II. Stimulation of P388D1, IC21, B16, L929 and Renca cells with IL-10 did not result in significant increase or decrease of *Ma* mRNA expression as compared to untreated control cells. Most interestingly, neither IL-4, IL-10 nor IFN-γ was able to modulate *Ma* mRNA expression in CIITA- and MHC class II-negative cell lines including the mammary adenocarcinoma TS/A, the colon adenocarcinoma MC-38, the hepatic fibrosarcoma MCA-102 and the T cell-derived cell lines RMA, RMAs, EL-4 and YAC-1. However, both IL-4 and IL-10 markedly induced *Ma* mRNA levels in P815 mastocytoma cells which lack detectable *MHC* class II and *CIITA* mRNA expression (Table 2).

Taken together, these results indicate that H2-M, independent of CIITA, is constitutively expressed and that CIITA appears to be required for coordinate regulation of H2-M and MHC class II expression by cytokines that control Th1 or Th2 immune responses.

### *Mb1* and *Mb2* are differentially expressed in IL-4-, IL-10- or IFN-γ-stimulated tumour cells

We investigated whether *Mb1* and *Mb2* genes are differentially expressed in IL-4, IL-10 or IFN-γ-treated cells by ratio RT-PCR analysis. Discrimination between co-amplified *Mb1* and *Mb2* isoforms was performed by restriction enzyme analysis using specific restriction sites for each *Mb* type in the amplified DNA (see Materials and Methods section). In summary, a divergent expression pattern of both *Mb* genes could be observed (Table 3). *Mb2* was predominantly expressed in the transformed B cell lines A20 and LB27.4. As shown in Figure 2A, the majority of the $Mb^d$ amplicons from untreated, IL-4-, IL-10- or IFN-γ-stimulated A20 B cells was cleaved by the $Mb2^d$-specific restriction endonuclease *XbaI* (Figure 2A, lane X). This result is confirmed by complementary digestion

© 2000 Cancer Research Campaign

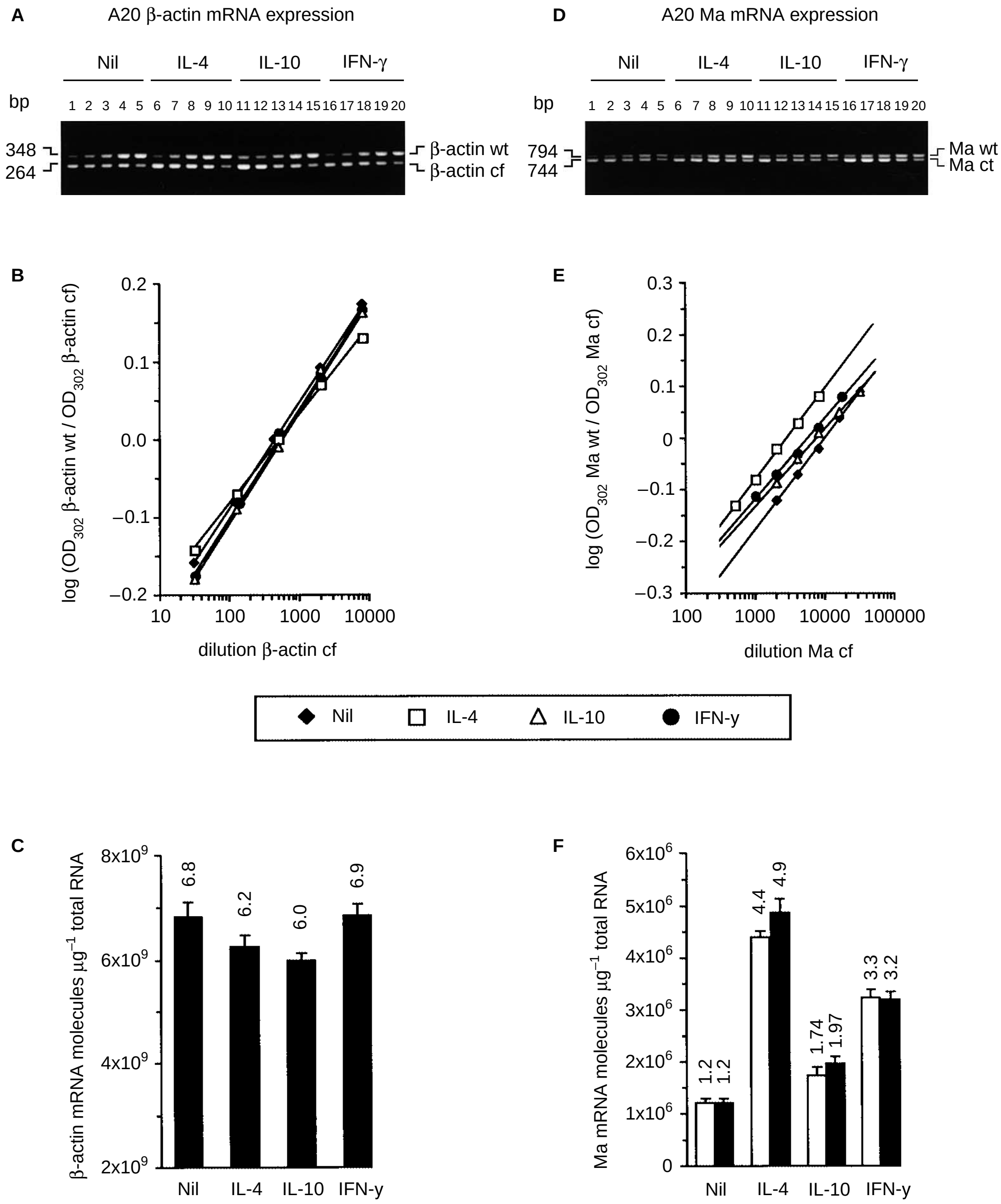

**Figure 1** Competitive RT-PCR analysis of *β-actin* and *H2-Ma* mRNA expression in IL-4, IL-10 or IFN-γ-stimulated A20 B cells. A20 cells were cultured in medium alone (Nil) or medium supplemented with 250 U ml$^{-1}$ IL-4, 100 ng ml$^{-1}$ IL-10 or 150 U ml$^{-1}$ IFN-γ for 48 h prior to RNA isolation and cDNA synthesis. *Left panel*, evaluation of *β-actin* mRNA concentration in each cDNA sample. (**A**) PCR reactions were performed by titrating 4-fold serial dilutions of the β-actin competitor plasmid, starting with $4.8 \times 10^9$ molecules (in lanes 1, 7, 11 and 16) with a constant amount of cDNA (50 ng). After 30 amplification cycles, PCR products were resolved on a 1.7% agarose/EtBr gel and intensity of each band corresponding to the cellular β-actin amplicon (wt) and β-actin competitor fragment (cf) was quantified as $OD_{302}$ units by densitometric imaging. (**B**) The log of the ratios of the two types of products were graphed as a function of the initial amount of competitor plasmid added to the PCR reactions. The initial amount of cellular *β-actin* mRNA in each reaction was determined by linear regression analysis and extrapolated from the point of the graph where the ratio of the two types of products is equimolar (log wt/cf = 0). (**C**) The specific *β-actin* mRNA levels for each sample expressed as mRNA molecules µg$^{-1}$ total cellular RNA. *Right panel*, estimation of *Ma* mRNA expression in IL-4, IL-10 or IFN-γ-treated A20 cells. (**D–E**) the same experiment as for the quantification of *β-actin* mRNA was performed, except that 100 ng cDNA of each sample and a 2-fold serial diluted Ma competitor fragment was used, starting with $5.86 \times 10^5$ molecules (for Nil and IL-10, lanes 1 and 11), $2.34 \times 10^6$ molecules (for IL-4, lane 6) and $1.17 \times 10^6$ molecules (for IFN-γ, lane 16). (**F**) The level of *Ma* mRNA expression in each sample was plotted as *Ma* mRNA molecules µg$^{-1}$ total cellular RNA before (open bars) and after normalization of their cDNA contents using *β-actin* mRNA expression as a standard (filled bars)

with *EaeI*, that specifically cleaves *Mb1*$^d$ (Figure 2A, lane E). As an additional control, complete cleavage was obtained by a combination of *XbaI* and *EaeI* (Figure 2A, lane E and X), demonstrating that the *Mb1* and *Mb2* expression pattern was not due to heterodimer formation, which may occur between closely related sequences during RT-PCR (Becker Andre and Hahlbrock, 1989). In comparison, both *Mb* genes were found to be expressed in the P815 mastocytoma cell line (Table 3). However, stimulation of P815 cells with IL-10 augmented *Mb2* expression, while IL-4 or IFN-γ did not exert any effect on the *Mb1* or *Mb2* mRNA expression pattern.

 *© 2000 Cancer Research Campaign*

**Table 2** Regulation of Ma mRNA expression in professional and nonprofessional APCs by IL-4 IL-10 and IFN-γ

| Cells | Nil | IL-4 | IL-10 | IFNγ |
|---|---|---|---|---|
| A20 | 1.0 (1.2 × $10^6$) | 4.1 (4.9 × $10^6$) | 1.7 (2.0 × $10^6$) | 2.7 (3.2 × $10^6$) |
| LB27.4 | 1.0 (1.6 × $10^6$) | 6.1 (9.8 × $10^6$) | 1.5 (2.4 × $10^6$) | 2.9 (4.6 × $10^6$) |
| P388D1 | 1.0 (2.2 × $10^5$) | 2.7 (5.9 × $10^5$) | 0.9 (1.9 × $10^5$) | 5.9 (1.3 × $10^6$) |
| IC-21 | 1.0 (8.1 × $10^4$) | 3.5 (2.8 × $10^5$) | 0.9 (7.3 × $10^4$) | 11.2 (9.1 × $10^5$) |
| B16 | 1.0 (2.4 × $10^4$) | 1.1 (2.7 × $10^4$) | 1.0 (2.4 × $10^4$) | 10.4 (2.5 × $10^5$) |
| L929 | 1.0 (5.1 × $10^4$) | 1.4 (7.3 × $10^4$) | 1.2 (6.3 × $10^4$) | 6.5 (3.3 × $10^5$) |
| Renca | 1.0 (4.3 × $10^4$) | 1.1 (4.6 × $10^4$) | 1.1 (4.7 × $10^4$) | 3.0 (1.3 × $10^5$) |
| P815 | 1.0 (8.5 × $10^4$) | 3.0 (2.6 × $10^5$) | 3.8 (3.2 × $10^5$) | 1.3 (1.1 × $10^5$) |
| TS/A | 1.0 (8.6 × $10^3$) | 1.0 (8.8 × $10^3$) | 1.4 (1.2 × $10^4$) | 1.3 (1.1 × $10^4$) |
| MC-38 | 1.0 (2.5 × $10^4$) | 1.0 (2.4 × $10^4$) | 1.4 (3.5 × $10^4$) | 1.0 (2.6 × $10^4$) |
| MCA-102 | 1.0 (1.3 × $10^4$) | 1.2 (1.5 × $10^4$) | 1.0 (1.2 × $10^4$) | 1.2 (1.6 × $10^4$) |
| EL-4 | 1.0 (3.9 × $10^3$) | 1.2 (4.6 × $10^3$) | 0.9 (3.6 × $10^3$) | 1.0 (4.0 × $10^3$) |
| RMAs | 1.0 (1.1 × $10^4$) | 0.9 (9.8 × $10^3$) | 0.9 (1.0 × $10^4$) | 1.1 (1.2 × $10^4$) |
| RMA | 1.0 (4.3 × $10^4$) | 1.3 (5.5 × $10^4$) | 1.0 (4.4 × $10^4$) | 1.1 (4.8 × $10^4$) |
| YAC-1 | 1.0 (3.6 × $10^4$) | 1.3 (4.7 × $10^4$) | 1.1 (4.0 × $10^4$) | 0.9 (3.2 × $10^4$) |

Cells were cultured in medium (Nil) or medium supplemented with 250 U $ml^{-1}$ IL-4, 100 ng $ml^{-1}$ IL-10 or 150 U $ml^{-1}$ IFN-γ for 48 h prior to RNA isolation and cDNA synthesis. Expression of *Ma* mRNA was determined by competitive RT-PCR. *β-actin* mRNA expression was used to normalize each individual sample. Relative *Ma* mRNA expression in cytokine-treated samples of each cell line is expressed as the ratio: *Ma* mRNA in cytokine treated cells / Mα mRNA expression (ratio = 1.0) in untreated cells. The number of *Ma* mRNA molecules μ$g^{-1}$ total RNA for each sample are shown in brackets.

**Table 3** Differential expression of Mb1 and Mb2 mRNA in IL-4, IL-10 or IFNγ-treated professional and nonprofessional APCs

| | Nil | | IL-4 | | IL-10 | | IFNγ | |
|---|---|---|---|---|---|---|---|---|
| Cells | *Mb1* | *Mb2* | *Mb1* | *Mb2* | *Mb1* | *Mb2* | *Mb1* | *Mb2* |
| A20 | 4.3 ± 0.5 | 95.7 ± 0.4 | 5.9 ± 1.3 | 94.1 ± 0.9 | 14.6 ± 2.0 | 85.4 ± 1.7 | 6.7 ± 1.1 | 93.3 ± 0.7 |
| LB27.4 | 16.8 ± 2.5 | 83.2 ± 4.2 | 15.9 ± 1.6 | 84.1 ± 2.0 | 27.7 ± 2.4 | 72.3 ± 3.9 | 15.3 ± 1.7 | 84.7 ± 3.1 |
| P815 | 43.6 ± 1.7 | 56.4 ± 2.4 | 39.3 ± 2.1 | 60.7 ± 2.0 | 28.6 ± 2.8 | 71.4 ± 2.3 | 44.1 ± 1.1 | 55.9 ± 1.7 |
| P388D1 | 88.7 ± 0.3 | 11.3 ± 0.7 | 83.8 ± 1.3 | 16.2 ± 2.4 | 89.2 ± 1.2 | 10.8 ± 0.9 | 93.6 ± 0.2 | 6.4 ± 0.6 |
| TS/A | 64.5 ± 1.3 | 35.5 ± 1.4 | 63.6 ± 2.0 | 36.4 ± 1.5 | 67.8 ± 2.8 | 32.2 ± 1.8 | 97.3 ± 0.9 | 2.7 ± 0.6 |
| Renca | 82.7 ± 2.5 | 17.3 ± 4.2 | 84.8 ± 3.9 | 15.2 ± 4.5 | 88.4 ± 1.7 | 11.6 ± 2.5 | 91.2 ± 3.6 | 8.8 ± 2.4 |
| MC-38 | 100 | 0 | 100 | 0 | 100 | 0 | 100 | 0 |
| IC-21 | 100 | 0 | 100 | 0 | 100 | 0 | 100 | 0 |
| B16 | 100 | 0 | 100 | 0 | 100 | 0 | 100 | 0 |
| L929 | 100 | 0 | 100 | 0 | 100 | 0 | 100 | 0 |
| MCA-102 | 100 | 0 | 100 | 0 | 100 | 0 | 100 | 0 |
| EL-4 | 100 | 0 | 100 | 0 | 100 | 0 | 100 | 0 |
| RMA | 100 | 0 | 100 | 0 | 100 | 0 | 100 | 0 |
| RMAs | 100 | 0 | 100 | 0 | 100 | 0 | 100 | 0 |
| YAC-1 | 100 | 0 | 100 | 0 | 100 | 0 | 100 | 0 |

Expression of *Mb1* and *Mb2* mRNA was assayed by ratio-RT-PCR analysis. Cells were cultured in medium (Nil) or medium supplemented with 250 U $ml^{-1}$ IL-4, 100 ng $ml^{-1}$ IL-10 or 150 U $ml^{-1}$ IFN-γ for 48 h prior to assay. Values indicate the relative expression levels of alternative *Mb* isoforms as a percentage of total *Mb* mRNA expression. Values are means ± SD for three determinations.

In contrast, *Mb1* was the prominent transcript in P388D1 macrophages and in tumour cells of epithelial origin including the renal adenocarcinoma Renca and the mammary adenocarcinoma TS/A. IFN-γ-treatment of P388D1, Renca and TSA cells enhanced *Mb1* expression as compared to untreated, IL-4- or IL-10-stimulated cells. Moreover, *Mb1* was found to be exclusively expressed in IC-21 macrophages, L929 fibroblasts, the hepatic fibrosarcoma MCA 102, the colon adenocarcinoma MC-38 and the B16 melanoma, as well as in the T cell-derived cell lines RMA, RMAs, EL-4 and YAC-1 (Table 3). As shown in Figure 2B, *Bam*HI digestion of $Mb^b$ amplicons from IL-4-, IL-10- or IFN-γ-treated B16 and L929 cells yielded restriction fragments exclusively corresponding to $Mb1^b$ transcripts, while fragments corresponding to $Mb2^b$ could not be detected as compared to the C57/BL ($H2^b$) spleen control. The complete cleavage of the $Mb^b$ amplification product by *Bam*HI confirmed that heterodimer formation did not occur during RT-PCR amplification.

### Mαβ1 and Mαβ2 heterodimers are expressed in tumour cells

Next, we addressed the question whether differential expression of *Ma*, *Mb1* and *Mb2* genes is restricted to mRNA expression, or whether Mαβ1 and Mαβ1 heterodimers exist in tumour cells of different histology. Western immunoblots of lysates from untreated, IL-4-, IL-10- or IFN-γ-stimulated A20 B cells, P388D1 macrophages, B16 melanoma and EL-4 thymoma cells were stained with a polyclonal rabbit serum raised against the cytoplasmic domain of Mα or an antiserum which recognizes both Mβ isoforms in order to assess H2-M protein expression (Figure 3). As expected (see Table 2), A20, P388D1, B16 and EL-4 cells did express detectable amounts of Mα monomers (Figure 3, left panel). Modulation of Mα monomer expression by IL-4, IL-10 or IFN-γ correlated with *Ma* mRNA expression levels in cytokine-

© 2000 Cancer Research Campaign 

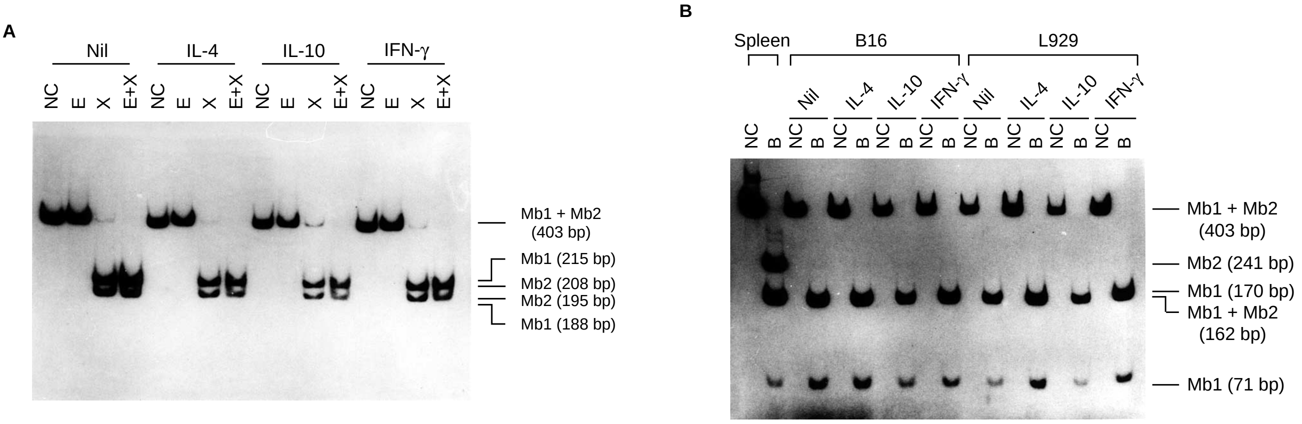

**Figure 2** Determination of *Mb1* and *Mb2* expression pattern in IL-4-, IL-10- or IFN-γ-treated A20 B cells (*H2$^d$*), B16 melanoma cells (*H2$^b$*) and transformed L929 fibroblasts (*H2$^b$*) by ratio-RT-PCR. Reverse transcriptase-PCR optimized for co-amplification of *Mb1* and *Mb2* transcripts, was performed in the presence of [α-$^{32}$P]dCTP as described in Materials and Methods. Co-amplified *Mb* isoforms were discriminated by restriction enzyme analysis followed by separation on 6% polyacrylamide gels. The length (bp) of the respective PCR products and the restriction fragments corresponding to *Mb1* or *Mb2* are indicated on the side of each figure. (**A**) *Mb1$^d$*/2$^d$ expression pattern in A20 cells. Lanes NC indicate the undigested PCR product; lanes E, show the *Mb1$^d$*-specific fragments obtained after digestion with *Eae*I; lanes X, the *Mb2$^d$*-specific fragments obtained after *Xba*I digest; lanes *E-X*, the total digestion by a combination of both enzymes. (**B**) *Mb1$^b$*/2$^b$ expression pattern in B16 and L929 cells. Splenocytes from C57BL/6 (*H2$^b$*) mice served as a positive control in this assay. Lanes NC show the undigested PCR product; lanes B, the *Mb1$^b$*- and *Mb2$^b$*-specific fragments obtained after *Bam*HI digestion. Where indicated, cells were cultured in medium alone (Nil) or in medium supplemented with 250 U ml$^{-1}$ IL-4, 100 ng ml$^{-1}$ IL-10 or 150 U ml$^{-1}$ IFN-γ for 48 h prior to RNA isolation and RT-PCR

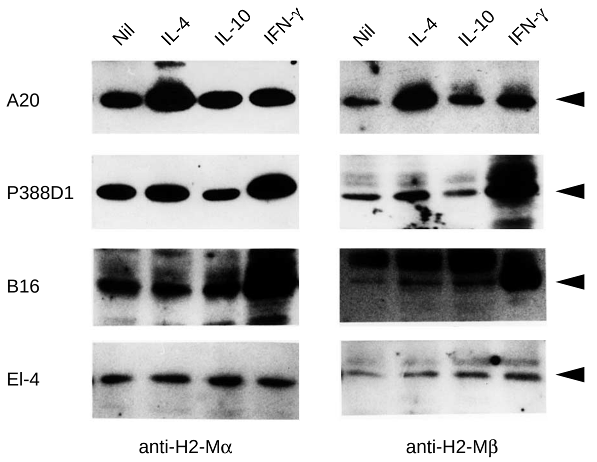

**Figure 3** H2-M protein expression is differentially regulated in A20 B cells, P388D1 macrophages, B16 melanoma cells and EL-4 thymoma cells by IL-4, IL-10 or IFN-γ. Whole cell lysates were separated using a denaturing SDS-PAGE (12.5%) and analysed by a Western immunoblot by staining with the rabbit antiserum R.Mα-C.69.3 (anti-Mα) or Mβ1/2-C.71.3 (anti-Mβ1 and -Mβ2). The arrowheads show the position of the ~ 27 kD Mβ monomers according to the predicted MW (Cho et al, 1991). Where indicated, cells were cultured in medium (Nil) or in medium supplemented with 250 U ml$^{-1}$ IL-4, 100 ng ml$^{-1}$ IL-10 or 150 U ml$^{-1}$ IFN-γ for 48 h before preparation of the cell lysates stimulated A20, P388D1, B16 and EL-4 cells (Table 2), suggesting that expression of *H2-M* gene expression is primarily regulated at the transcriptional level. Noteworthy, untreated, IL-4-, IL-10- or IFN-γ-stimulated P388D1, B16 and EL-4 cells expressed Mβ1 monomers, while A20 cells expressed Mβ2 (Table 3 and Figure 3, right panel). Comparison of Mα and Mβ expression in untreated, IL-4-, IL-10- or IFN-γ-stimulated A20, P388D1, B16 and EL-4 cells revealed a similar pattern, implicating that expression of *Ma* and *Mb* genes might be co-regulated similarly to human *DMA* and *DMB* (Peleraux et al, 1996).

### Alternative H2-M isoforms display similar functional activities in antigen presentation to H2-A$^d$- and H2-E$^d$-restricted CD4+ T cells

To assess whether differential expression of H2-M isoforms impacts on the presentation of native protein antigens, we studied the ability of Mαβ1-expressing P388D1 macrophages and Mαβ2-expressing A20 B cells to effectively process and present surrogate target antigens, ovalbumin (OVA) and influenza haemagglutinin (HA) and the corresponding peptides to H2-A$^d$- and H2-E$^d$-restricted CD4 + T cells from TCR transgenic mice. To ensure that levels of H2-M and MHC class II expressed in P388D1 macrophages were comparable to those in A20 B cells (Tables 1 and 2), P388D1 cells were stimulated with IFN-γ for 48 h prior to antigen presentation. As shown in Figure 4, P388D1 (Mαβ1) as well as A20 (Mαβ2) cells were able to process and present native OVA and HA to H2-A$^d$- and H2-E$^d$-restricted CD4+ T cells, respectively. In contrast, presentation of native antigens by fixed APCs was severely impaired, indicating that the capacity of P388D1 and A20 cells to process and present the $OVA_{323-339}$ and $HA_{107-122}$ determinants did not result from extracellular antigen processing or peptide contamination. As expected, native and paraformaldehyde-fixed P388D1 and A20 cells present exogenously supplied $OVA_{323-339}$ or $HA_{107-122}$ peptides to CD4+ T cell populations. Overall, these findings implicate equivalent functional capabilities for individual H2-M isoforms in selecting the $OVA_{323-339}$ or $HA_{107-122}$ T cell epitopes presented by H2-A$^d$ and H2-E$^d$, respectively.

### DISCUSSION

H2-M is a MHC class II-like heterodimeric molecule which appears to be required for efficient antigen presentation by many MHC class II alleles (Denzin et al, 1994; Miyazaki et al, 1996; Wolf et al, 1998). The rationale of this work was twofold, first to investigate whether *H2-M* and *MHC* class II genes are coordinately regulated by cytokines in tumour cells, and second, whether

*© 2000 Cancer Research Campaign*

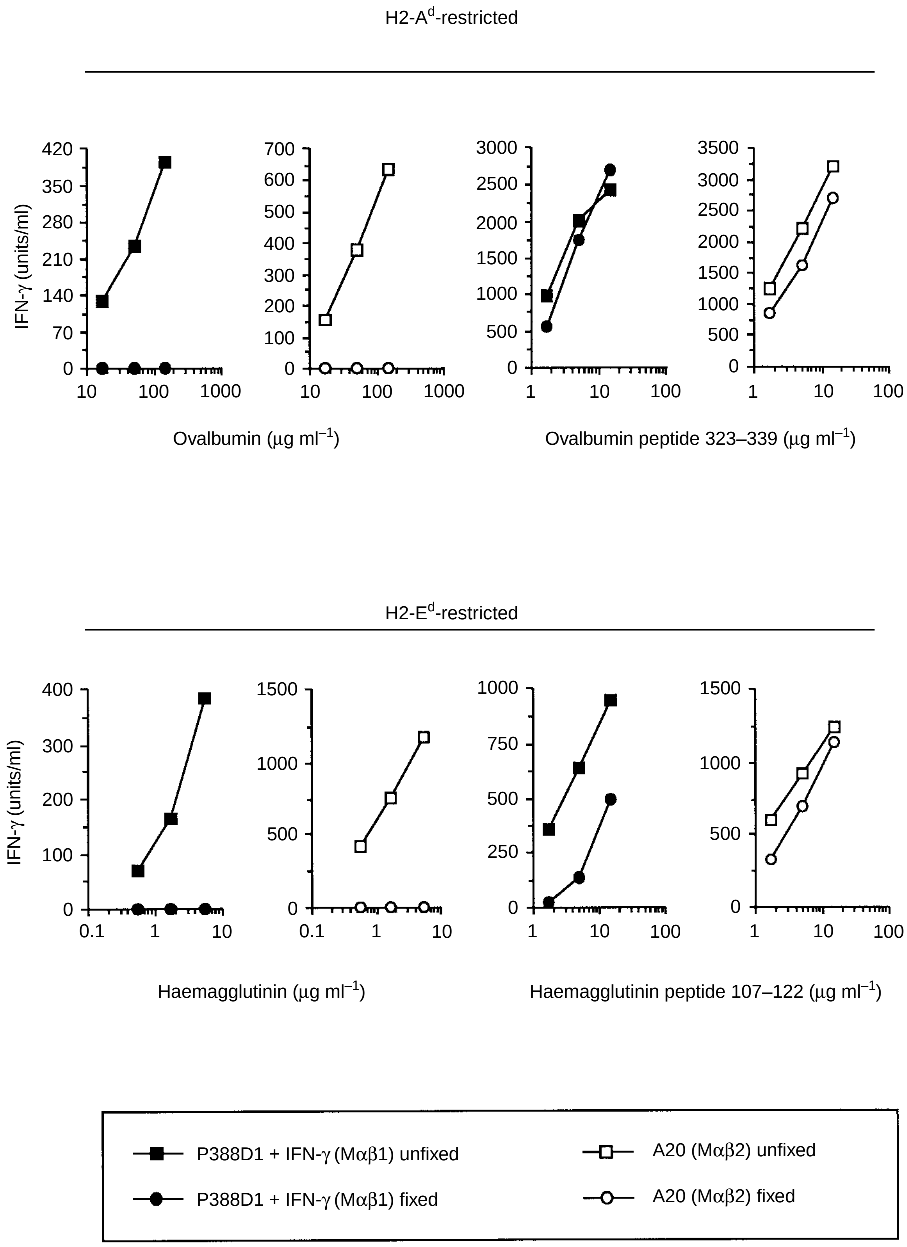

**Figure 4** APCs expressing Mαβ1 or Mαβ2 can efficiently process and present OVA and HA to H2-A$^d$. and H2-E$^d$-restricted CD4+ T cells. 2 × $10^5$ Th1 differentiated CD4+ Th cells (Lingnau et al, 1998) from TCR transgenic mice either specific for peptides 323–339 derived from OVA and presented by H2-A$^d$ (Murphy et al, 1990) or specific for peptides 111–119 from HA and presented by H2-E$^d$ (Kirberg et al, 1994) were cultured with 5 × $10^4$ IFN-γ-stimulated P388D1 (Mαβ1) or A20 cells (Mαβ2) as APCs in the presence of increasing antigen concentrations or synthetic peptides. Supernatants were collected after 24 h and tested for IFN-γ secretion. APCs were fixed with 1% paraformaldehyde prior to assay the two *Mb* genes of the *H2-M* region are differentially expressed and display similar or distinct functional activities in MHC class II antigen presentation.

The present study demonstrates that *H2-M* and *MHC* class II gene expression is coordinately regulated by IL-4, IL-10 or IFN-γ in transformed cells derived from professional APCs (i.e. macrophages or B cells) in the presence of CIITA. This is in agreement with previous data which indicated that the 5′ proximal promoter region of *H2-M* and *MHC* class II genes share conserved *cis*-acting sequences (Peleraux et al, 1996), particularly the ‘X1 box’, which binds the RFX transcription factor complex that appears to be crucial for CIITA interaction (Scholl et al, 1997).

In contrast, our analysis identified low levels of *H2-M* transcripts in nonprofessional APCs prior to IFN-γ-mediated induction of CIITA expression (Table 2), implicating that basal *H2-M* expression might occur independently of CIITA. Indeed, *CIITA*-deficient mice still exhibit a basal level of *Mb* expression in splenocytes (Chang et al, 1996), and some IFN-γ-responsive human cell lines express detectable amounts of *DM* transcripts in the absence of CIITA expression (Westerheide et al, 1997; Sartoris et al, 1998). Furthermore, our data demonstrate that murine tumour cell lines which are deficient in IFN-γ-mediated CIITA expression, such as MCA-102, TS/A, or MC-38 (Tables 1 and 2), do not exhibit increased basal *H2-M* expression levels, nor induction of *MHC* class II gene expression following IFN-γ-treatment. Taken together, these observations can be reconciled with a model in which CIITA is a prerequisite in professional and nonprofessional APCs for co-regulation of genes which are instrumental for efficient MHC class II antigen presentation. However, based on data presented in this report, CIITA might be no longer viewed as the exclusive regulator for *H2-M* expression.

Additionally, suppression of CIITA activity, in addition to the occurrence of tumour antigen loss variants (Kerkmann-Tucek et al, 1998), may provide one potential mechanism for neoplastic cells to escape immune surveillance. Notably not only the lack of inducible CIITA expression (e.g. tumour cells summarized in Table 1), may account for insufficient MHC class II cell surface expression. Other, as yet poorly defined, factors may be responsible for the failure of tumour cells to upregulate MHC class II. For instance, IFNγ-treatment of the renal cell carcinoma Renca results in *CIITA* mRNA expression (Table 1), enhanced *H2-Ma* mRNA expression, but does not lead to H2-A, or H2-E cell surface expression (Table 1).

*H2-Ma*, *-Mb1* and *-Mb2* are co-expressed in splenocytes of mice carrying different haplotypes (Walter et al, 1996), indicating that Mαβ1 and Mαβ2 heterodimers might be operational in antigen presentation by many MHC class II alleles/isotypes. As shown in this study, *Mb1* is predominantly expressed in transformed macrophages, melanoma cells and in tumour cells of epithelial and mesenchymal origin, while *Mb2* is expressed in B cells. Of note, the mastocytoma cell line P815 was found to express both *Mb* genes at almost equal levels (Table 3). Differential expression of members of a gene family in different cell types or at different developmental stages has been described for many eukaryotic genes (Hardison, 1998). For instance, an inverse relationship between gene activity and levels of CpG dinucleotide methylation has been observed (Hsieh, 1997; Agarwal and Rao, 1998). The 5′ flanking promoter region of both *Mb* genes contains conserved S, X1, X2 and Y elements (Peleraux et al, 1996) which is required for constitutive and inducible expression (Mach et al, 1996). Since DNA methylation has been proposed to affect chromatin structure (Hsieh, 1997), methylation of CpG islands may critically influence the binding of transcription factors to *cis*-acting sequences and which may be able to silence the transcriptional activity of the *Mb2* promoter in a cell-type-specific manner. Alternatively, but not mutually exclusive, cellular diversity of *Mb1* and *Mb2* expression might be controlled by cell-type-specific transcriptional activators selectively interacting with one of the *Mb* promoters. Alternative CIITA isoforms have been discovered, which selectively control cell-type-specific and inducible MHC class II expression (Muhlethaler Mottet et al, 1997).

*Mb1* and *Mb2* differ predominantly within exon 2, where similarity at the nucleotide level drops to 90%, as compared to 97–100% within the other exons (Walter et al, 1996). The present study addressed the question whether H2-M isoforms differ within their functional capabilities in MHC class II antigen presentation. P388D1 and A20 cells, which selectively express Mαβ1 and Mαβ2, respectively, can process and present the $OVA_{323-339}$ and $HA_{107-122}$ epitopes to H2-$A^d$- and H2-$E^d$-restricted CD4 + T cells, implicating a similar biological activity of both H2-M isoforms in mice carrying two functional MHC class II isotypes. Consistent with these observations, recent crystallographic analysis revealed a comparable overall molecular surface architecture of both H2-M isoforms, suggesting similar functional activities in peptide loading (Fremont et al, 1998).

The apparent lack or need of certain human or murine MHC class II alleles to require DM/H2-M for CLIP removal in vivo (Denzin et al, 1994; Miyazaki et al, 1996; Wolf et al, 1998) has been attributed to the affinities of these MHC class II alleles for CLIP as determined by in vitro binding studies (Sette et al, 1995). Moreover, the apparent lack of Ii or H2-M in tumour cells in the presence of MHC class II cell surface expression may turn out to be beneficial for the host. Tumour cells transfected with syngeneic MHC class II genes without co-expressing Ii or Ii/H2M are highly immunogenic and appear to present rather endogenous as compared to exogenous antigens to tumour-specific T cells (Armstrong et al, 1997).

In summary, Th1- and Th2-associated cytokines coordinately regulate expression of genes involved in the MHC class II antigen processing and presentation pathway in the presence of CIITA in a cell-type-specific manner. Yet, our findings established for the first time that Mαβ1 and Mαβ2 are selectively expressed in different murine tumour cells and that both isoforms can select for MHC class II/peptide assembly. Future studies may address whether differences in the MHC class II antigen presentation pathway in tumour cells impacts on disease progression and clinical outcome of vaccine strategies targeting tumour-associated antigens displayed by MHC class II molecules.

### ACKNOWLEDGEMENTS

The authors wish to thank K Freitag and C Neukirch for excellent technical assistance. This work was supported by the Deutsche Forschungsgemeinschaft (DFG), SFB 311/A16 and SFB 432/A9.

### REFERENCES

Agarwal S and Rao A (1998) Modulation of chromatin structure regulates cytokine gene expression during T cell differentiation. *Immunity* **9**: 765–775

Armstrong TD, Clements VK, Martin BK, Ting JPY and Ostrand-Rosenberg S (1997) Major histocompatibility complex class II-transfected tumour cells present endogenous antigen and are potent inducers of tumour-specific immunity. *Proc Natl Acad Sci USA* **94**: 6886–6891

Bakke O and Dobberstein B (1990) MHC class II-associated invariant chain contains a sorting signal for endosomal compartments. *Cell* **63**: 707–716

Becker Andre M and Hahlbrock K (1989) Absolute mRNA quantification using the polymerase chain reaction (PCR). A novel approach by a PCR aided transcript titration assay (PATTY). *Nucleic Acids Res* **17**: 9437–9446

Bouaboula M, Legoux P, Pessegue B, Delpech B, Dumont X, Piechaczyk M, Casellas P and Shire D (1992) Standardization of mRNA titration using a polymerase chain reaction method involving co-amplification with a multispecific internal control. *J Biol Chem* **267**: 21830–21838

Chang CH, Guerder S, Hong SC, van Ewijk W and Flavell RA (1996) Mice lacking the MHC class II transactivator (CIITA) show tissue-specific impairment of MHC class II expression. *Immunity* **4**: 167–178

Cho SG, Attaya M and Monaco JJ (1991) New class II-like genes in the murine MHC. *Nature* **353**: 573–576

Cresswell P (1996) Invariant chain structure and MHC class II function. *Cell* **84**: 505–507

Cresswell, P (1998) Proteases, processing, and thymic selection. *Science* **280**: 394–395

Denzin LK, Hammond C and Cresswell P (1996) HLA-DM interactions with intermediates in HLA-DR maturation and a role for HLA-DM in stabilizing empty HLA-DR molecules. *J Exp Med* **184**: 2153–2165

 *© 2000 Cancer Research Campaign*

Denzin LK, Robbins NF, Carboy Newcomb C and Cresswell P (1994) Assembly and intracellular transport of HLA-DM and correction of the class II antigen-processing defect in T2 cells. *Immunity* **1**: 595–606
Fremont DH, Crawford F, Marrack P, Hendrickson WA and Kappler J (1998) Crystal structure of mouse H2-M. *Immunity* **9**: 385–393
Haberhausen G, Pinsl J, Kuhn CC and Markert Hahn C (1998) Comparative study of different standardization concepts in quantitative competitive reverse transcription-PCR assays. *J Clin Microbiol* **36**: 628–633
Hardison R (1998) Hemoglobins from bacteria to man: evolution of different patterns of gene expression. *J Exp Biol* **201**: 1099–1117
Hsieh CL (1997) Stability of patch methylation and its impact in regions of transcriptional initiation and elongation. *Mol Cell Biol* **17**: 5897–5904
Kelly AP, Monaco JJ, Cho SG and Trowsdale J (1991) A new human HLA class II-related locus, DM. *Nature* **353**: 571–573
Kerkmann-Tucek A, Banat GA, Cochlovius B and Zoller M (1998) Antigen loss variants of a murine renal cell carcinoma: implications for tumour vaccination. *Int J Cancer* **77**: 114–122
Kirberg J, Baron A, Jakob S, Rolink A, Karjalainen K and von Boehmer H (1994) Thymic selection of CD8+ single positive cells with a class II major histocompatibility complex-restricted receptor. *J Exp Med* **180**: 25–34
Kleijmeer MJ, Morkowski S, Griffith JM, Rudensky AY and Geuze HJ (1997) Major histocompatibility complex class II compartments in human and mouse B lymphoblasts represent conventional endocytic compartments. *J Cell Biol* **139**: 639–649
Lingnau K, Hoehn P, Kerdine S, Koelsch S, Neudoerfl C, Palm N, Ruede E and Schmitt E (1998) IL-4 in combination with TGF-beta favors an alternative pathway of Th1 development independent of IL-12. *J Immunol* **161**: 4709–4718
Mach B, Steimle V, Martinez Soria E and Reith W (1996) Regulation of MHC class II genes: lessons from a disease. *Annu Rev Immunol* **14**: 301–331
Miyazaki T, Wolf P, Tourne S, Waltzinger C, Dierich A, Barois N, Ploegh H, Benoist C and Mathis D (1996) Mice lacking H2-M complexes, enigmatic elements of the MHC class II peptide-loading pathway. *Cell* **84**: 531–541
Mongini C, Sanchez Lockart M, Waldner CI, Alvarez EM, Gravisaco MJ, Roig MI and Hajos SE (1996) Enhancement of anti-tumour immunity in syngeneic mice after MHC class II gene transfection. *Br J Cancer* **74**: 258–263
Muhlethaler Mottet A, Otten LA, Steimle V and Mach B (1997) Expression of MHC class II molecules in different cellular and functional compartments is controlled by differential usage of multiple promoters of the transactivator CIITA. *EMBO J* **16**: 2851–2860
Murphy KM, Heimberger AB and Loh DY (1990) Induction by antigen of intrathymic apoptosis of CD4+ CD8+ TCRlo thymocytes in vivo. *Science* **250**: 1720–1723
Ossendorp F, Mengede E, Camps M, Filius R and Melief CJ (1998) Specific T helper cell requirement for optimal induction of cytotoxic T lymphocytes against major histocompatibility complex class II negative tumours. *J Exp Med* **187**: 693–702
Pardoll DM and Topalian SL (1998) The role of CD4+ T cell responses in antitumour immunity. *Curr Opin Immunol* **10**: 588–594
Peleraux A, Karlsson L, Chambers J and Peterson PA (1996) Genomic organization of a mouse MHC class II region including the H2-M and Lmp2 loci. *Immunogenetics* **43**: 204–214
Riberdy JM, Newcomb JR, Surman MJ, Barbosa JA and Cresswell P (1992) HLA-DR molecules from an antigen-processing mutant cell line are associated with invariant chain peptides. *Nature* **360**: 474–477
Sanderson F, Thomas C, Neefjes J and Trowsdale J (1996) Association between HLA-DM and HLA-DR in vivo. *Immunity* **4**: 87–96
Sartoris S, Valle MT, Barbaro AL, Tosi G, Cestari T, D'Agostino A, Megiovanni AM, Manca F and Accolla RS (1998) HLA class II expression in uninducible hepatocarcinoma cells after transfection of AIR-1 gene product CIITA: acquisition of antigen processing and presentation capacity. *J Immunol* **161**: 814–820
Scholl T, Mahanta SK and Strominger JL (1997) Specific complex formation between the type II bare lymphocyte syndrome-associated transactivators CIITA and RFX5. *Proc Natl Acad Sci USA* **94**: 6330–6334
Sette A, Southwood S, Miller J and Appella E (1995) Binding of major histocompatibility complex class II to the invariant chain-derived peptide, CLIP, is regualted by allelic polymorphism in class II. *J Exp Med* **181**: 677–683
Urban RG, Chicz RM and Strominger JL (1994) Selective release of some invariant chain-derived peptides from HLA-DR1 molecules at endosomal pH. *J Exp Med* **180**: 751–755
Van Ham SM, Gruneberg U, Malcherek G, Broker I, Melms A and Trowlsdale J (1996) Human histocompatibility leukocyte antigen (HLA)-DM edits peptides presented by HLA-DR according to their ligand binding motifs. *J Exp Med* **184**: 2019–2024
Walter W, Loos M and Maeurer MJ (1996) H2-M polymorphism in mice susceptible to collagen-induced arthritis involves the peptide binding groove. *Immunogenetics* **44**: 19–26
Walter W, Scheuer C, Loos M, Reichert TE and Maeurer MJ. H2-Mβ1 or H2-Mβ2 heterodimers equally promote CLIP removal in 1-$A^q$ molecules from autoimmune-prone DBA/1 mice. *J Biol Chem* (in press)
Westerheide SD, Louis Plence P, Ping D, He XF and Boss JM (1997) HLA-DMA and HLA-DMB gene expression functions through the conserved S-X-Y region. *J Immunol* **158**: 4812–4821
Wolf PR and Ploegh HL (1995) How MHC class II molecules acquire peptide cargo: biosynthesis and trafficking through the endocytic pathway. *Annu Rev Cell Dev Biol* **11**: 267–306
Wolf PR, Tourne S, Miyazaki T, Benoist C, Mathis D and Ploegh HL (1998) The phenotype of H-2M-deficient mice is dependent on the MHC class II molecules expressed. *Eur J Immunol* **28**: 2605–2618

© 2000 Cancer Research Campaign
